# Consumption

**Published:** 1891-03

**Authors:** C. A. Gill

**Affiliations:** Madison, Wisconsin


### CONSUMPTION.

BY C. A. GILL, M. D., MADISON, WISCONSIN.*

Tuberculosis is a disease of the highest importance, and calculated to excite a very deep interest, from the fact that it is so wide-spread, scarcely a spot of the inhabitable globe being exempt from its ravages, from the character of its victims and from its being so intractable to treatment. It respects neither age, sex or condition. It invades the hovels of the poor and the palaces of the rich. Most persons die of consumption in the bloom of youth, at a period when hopes are brightest, and the capacities of enjoying life are in full vigor and maturity. Most of its victims are remarkable for the early unfolding and brilliancy of their mental accomplishments. And many a family has to regret that by tuberculosis some of the fairest and best of its members have been hurried to an early grave. The disease has always occupied a prominent place in the history of medicine from the time of Hippocrates, Araetus and Galen to the present time. The term *tubercle*, however, did not have the significance at that time that it has at present. At that time it was used in its anatomical sense, signifying an induration, a nodosity. At that time also any disease within the chest accompanied with an accumulation of pus was considered consumption. The physicians of that time concluded that it was due to an ordinary inflammation, without

* Read before the Central Wisconsin Medical Society.

there being anything specific in its nature. This theory obtained for the period of eighteen hundred years or until the time of Bayle, who now came forward with the theory that tuberculosis was a specific disease, which he arrived at from repeated post-mortem examinations, and these could be pursued impunity only until about the seventeenth century, when he declared that tubercular phthisis was not a disease produced by inflammation of the lungs, pleurae or bronchi. These affections may complicate or precipitate a fatal result, but they never constitute its cause. Had succeeding clinicians and pathologists been content to stand by the opinion of Bayle, our present knowledge of the pathology of the disease might have been advanced half a century. Bayle, however, knew of no signs by which the disease could be detected during life. This remained for Laennec to discover by auscultation. This was purely and solely the invention of Laennec, as there had been no previous discoveries in this direction. The observations of Bayle and Laennec were confirmed by Louis in 1825 by a study of 193 cases of the disease and would seem to have finally settled the question of the specific nature of the disease. But no, in 1847 Virchow, one of the most distinguished pathologists of the age, succeeded in overthrowing to a certain extent the belief in the specific character of the disease and thus relegating it to the same position that it occupied in the time of Hippocrates and Galen. But the rank and file of the profession followed in the main the views of Laennec, as they harmonized so with clinical experience. The year 1865 marks an important epoch in the history of tuberculosis. At this time Villeman advanced the views of the inoculability of the disease. Villeman concluded from his experiments : (1) Tuberculosis is a specific disease. (2) It is inoculable. (3) It may be successfully inoculated in rabbits from man. (4) It belongs among the virulent affections and takes its place in nosology with small-pox, scarlet fever, syphilis, and glanders. The disease arises therefore by direct inoculation or by contagion. These experiments at once excited the greatest interest, and the Paris Academy of Medicine appointed a committee to report upon Villeman's work, and they confirmed his experiments. The specific nature of tuberculosis seemed to be permanently established; but experimenters far and wide commenced their injections of various substances and demonstrated that they could produce the same lesions, and this lent support to the views of pathologists that the disease might be caused by any kind of inflammation. Villeman's triumph was of short duration and within four or five years had been almost entirely ignored. But it was only for a short time, for in 1878 Tappeiner caused a number of dogs to inhale tuberculous sputa, and thereby caused the lesions of the disease to be produced in the lungs. These were only confirmations of the conclusions of Villeman. From this time on the doctrine of the specific nature of the disease gained adherents every day. Its specific nature once established, it only remained to discover the character of the virus. This professor Koch did in 1882, and forever immortalized his name.

In the preceding remarks, I have endeavored to give you a short synopsis of the history of tuberculosis. I shall briefly consider the treatment.

Our therapeutics of the disease have not kept pace with pathology. Many specifics have been vaunted, but none have stood the test of time and experience. The treatment falls naturally under four heads, viz., *hygienic*, *dietetic*, *climatic* and *medicinal*.

1. The hygienic surroundings of the patient must be as perfect as possible. He should lead an outdoor life exclusively. His sleeping room should admit plenty of fresh air at night, and during the day time the windows should be thrown wide open and the room thoroughly aired. He should practice chest breathing as much as possible without fatigue. Horse-back exercise is the exercise, *par excellence*, as many times a patient is too feeble to walk who can take considerable exercise on horse-back. Parenthetically, let me say, do not advise your patient to take horse-back exercises unless his finances are such as to enable him to own a horse. And so with all the rest of the treatment, let it be adapted to the condition of the patient. He should wear woolen under-clothing at all times and keep the skin active by a daily bath. At the present time it is pretty generally conceded that the disease is not transmitted from one generation to another, but only a predisposition. Bearing this in mind, we should see that every child of phthisical parents should be placed in the best possible hygienic surroundings and not allowed to occupy the same sleeping apartments with its parents, and see that it gets plenty of good nutritious food rich in hydro-carbons.

2. THE CLIMATIC TREATMENT.—The climate must be of an equable temperature. A high latitude is best as less likely to contain germs, and it is also drier, an important consideration, as I think the idea which the Grecian fathers held, viz., that a dry air had a dessicating effect on the ulcers, has an element of truth in it. The climate must be one in which there is plenty of ozone generated, which acts as a stimulant to the system, and at the same time lessens the production of germs. We should recollect, however, that no climate is a specific for consumption; and the physician should consider well and examine the question in all its bearings before sending his patient away from home and its comforts and conveniences separating him from family and friends and subjecting him to the discomforts and inconveniences of a strange place, with the depressing influence of home-sickness to further debilitate an already enfeebled system. It must be a source of regret to the physician who, without weighing well all of the factors in this problem of climate, sends his patient to Florida, Colorado or some other health resort to have him die in a few months among strangers, denied the many acts of kindness which only family ties can render possible. Volumes might be written on this question of climate, but I will not trespass upon your time any further in this direction.

3. DIETETICS. The food should consist of the most nutritious and easily digested articles of diet, such as raw beef, eggs, milk, fat in various forms, and vegetables. In many of these cases the stomach is in a catarrhal state and the secretions of the lower bowels need stimulating. We should correct these conditions as much as possible by siphoning out the stomach with sterilized water, or directing the patient to drink a glass of hot water a half hour or an hour before food. We should recollect that there is no remission of the physical sins except through the baptism of healthy blood, and to secure this the food, although taken in proper quantity and quality, will not accomplish unless the alimentary canal is in a condition to digest and assimilate it. Food should be taken frequently every three or four hours and at a time when the body is not exhausted. We should explain to patient the important role fats play in the treatment of consumption, and insist on their taking them in some form or other regardless of taste.

4. MEDICINAL TREATMENT. I believe that much good may result from the intelligent and discriminating use of drugs. But often, too often they are not administered in this way, the physician should weigh well the indications for the use of this or that drug and administer it accordingly. If there are night sweats give atropia, as I believe this is the drug *par excellence* for this condition. By checking the loss of the salts of the blood, the appetite, which before did not respond to tonics, will begin to improve and a decided change for the better take place. It is also an excellent respiratory stimulant. For tonics, iron, arsenic and strychnine take first place, and of these I myself place most reliance on arsenic. It is not only a tonic, but it is also an alterative, and an antiseptic. And right here let me add that Fothergill struck a keynote when he said that "when you find the tongue red and denuded of its epithelium withhold your tonics and instead give alkalies," a principle which I have found to hold true in practice. If there is cough give some simple cough mixture, but avoid morphia, unless in the latter stages of the disease for the fever sponge baths, avoiding antipyretics, if possible. There is one drug which I think is given too indiscriminately without due regard to the condition of the patient's digestive capacities, and that is cod liver oil. We have a very eligible preparation now in *Mollus*, which is devoid of most of the objectionable features of the older preparations and quite apt to agree with the most fastidious stomach. I am in the habit of giving alcohol in every case, usually combining it with cod liver oil after meals, as by giving it in this way there is less likely to be a habit established. It is a stimulant, a food, and an antiseptic. There is one drug that has come into considerable prominence of late and that is *creasote*. I have used the drug in several cases in connection with other treatment with decided benefit. Just what part the creasote played in the improvement, I can not say. The consensus of opinion of a large number of physicians who have given it extensive trial seems to be favorable to it. As to whether it interferes with the bacilli through the circulation by virtue of its antiseptic properties, or whether it favors general nutrition by its tonic qualities they are not decided.

In connection with this subject of tuberculosis I would like to report a case which came under my observation a short time ago, and which I treated. It is as follows:

CASE.—Mr. T., age 45, came to me for treatment August 2nd. Family history negative as regards lung trouble; previous health good until the February preceding, when he had an attack of *la Grippe*, from which he had never fully recovered. He had been treated by a physician for three weeks before coming to me for what he called pleurisy. His temperature was 101 degree, pulse 100, respiration 24. He had profuse night sweats, severe cough, dyspnoea on examination, and loss of appetite, all of the symptoms of phthisis you may say, and yet the physical signs were chiefly negative. There were a few crepitant rales at the upper part of both lungs with a friction murmur on the right side of the chest. I informed him that I thought he had consumption, but could not say positively until I had examined his sputum. I obtained a specimen and sent it to Professor Birge who examined and reported the result as negative. As the specimen was 48 hours old and had begun to decompose, he desired a fresh specimen, which I secured for him. As a result of the latter examination he wrote me that he had discovered tubercle bacilli to the number of 40 or 50 in each slide, and thus confirmed the suspicions which I had previously entertained.

If you will pardon the digression, I will say I think that physician derelict in his duty who does not exhaust every diagnostic means at his command to discover this widespread and most fatal disease at his earliest opportunity.

For his night sweats I gave him pills of atropia, gr. 1-60, at bedtime, which I had to increase to gr. 1-30, and I was able to check them. I prescribed creasote in two grain capsules, one after each meal, increasing one a day until he took seven. His appetite began to improve. He gained in strength, and there was a gradual and steady improvement in all his symptoms. His weight, which at the commencement was 120 pounds or 18 pounds less than his normal weight, began to improve, so that within six weeks from the commencement of treatment he had regained his original weight. He did not stop here, however, but continued to gain in weight and to-day weighs 156 pounds with not a particle of cough and apparently a well man. I forgot to state I also used alcohol and cod liver oil in the treatment. And so we see that consumption can be cured in its incipient stages without Koch's or Shurley's lymph. When we take into consideration the fact that one-half the people who die after reaching adult age show lesions of previous tuberculosis, it is evidence that the recuperative powers of the system alone are able to deal with this formidable foe at times.

As to Koch's lymph, it is too early as yet to express an opinion as to its merits. If it proves to be what has been claimed for it, Professor Koch has added another jewel to the crown which he already possesses as the greatest medical discoverer of the age. Let us hope for the afflicted multitudes that it may prove to be the long sought for specific.

